# Multiscale Experimental and Theoretical Investigations of Spin Crossover Fe^II^ Complexes: Examples of [Fe(phen)_2_(NCS)_2_] and [Fe(PM-BiA)_2_(NCS)_2_]

**DOI:** 10.3390/ijms16024007

**Published:** 2015-02-12

**Authors:** Samir F. Matar, Philippe Guionneau, Guillaume Chastanet

**Affiliations:** CNRS, Univ. Bordeaux, ICMCB, UPR 9048, F-33600 Pessac, France; E-Mails: philippe.guionneau@icmcb.cnrs.fr (P.G.); guillaume.chastanet@icmcb.cnrs.fr (G.C.)

**Keywords:** spin-crossover complex, DFT, photo-switching, molecular dynamics, (P, T) phase diagram

## Abstract

For spin crossover (SCO) complexes, computation results are reported and confirmed with experiments at multiscale levels of the isolated molecule and extended solid on the one hand and theory on the other hand. The SCO phenomenon which characterizes organometallics based on divalent iron in an octahedral FeN_6_-like environment with high spin (HS) and low spin (LS) states involves the LS/HS switching at the cost of small energies provided by temperature, pressure or light, the latter connected with Light-Induced Excited Spin-State Trapping (LIESST) process. Characteristic infra red (IR) and Raman vibration frequencies are computed within density functional theory (DFT) framework. In [Fe(phen)_2_(NCS)_2_] a connection of selected frequencies is established with an ultra-fast light-induced LS → HS photoswitching mechanism. In the extended solid, density of state DOS and electron localization function (ELF) are established for both LS and HS forms, leading to characterizion of the compound as an insulator in both spin states with larger gaps for LS configuration, while keeping molecular features in the solid. In [Fe(PM-BiA)_2_(NCS)_2_], by combining DFT and classical molecular dynamics, the properties and the domains of existence of the different phases are obtained by expressing the potential energy surfaces in a short range potential for Fe–N interactions. Applying such Fe–N potentials inserted in a classical force field and carrying out molecular dynamics (MD) in so-called “semi-classical MD” calculations, lead to the relative energies of HS/LS configurations of the crystal and to the assessment of the experimental (P, T) phase diagram.

## 1. Introduction

Within the large community of researchers involved with molecular sciences one major objective is to study compounds that have the ability to be photo-, piezo-, and/or thermo-stimulated. Such a research axis merges in the broad context of the development of candidate materials for incorporation in devices devoted to: the treatment of information and data storage; their use as smart X-chromic pigments; and to be envisaged as molecular engines or molecular switches. The key property of such stimulable molecular compounds is their “bi-stability”. This property characterizes a distinctive class of transition metal complexes of the first period (3*d*) exhibiting the ability of switching between two spin states of 3*d* electrons, the High Spin state (HS) and the Low Spin state (LS). The observed physical change is known as the Spin Cross-Over (SCO) phenomenon. It is mainly exhibited in divalent iron complexes where Fe^II^ (3*d*^6^) is embedded in an octahedral-like environment. The relevant physical feature is at the magnetic level where Fe^II^ switches between a diamagnetic Low Spin (LS, t_2g_^6^e_g_^0^) state with S = 0 and ^1^A_1g_ spectroscopic term, and a paramagnetic High Spin (HS, t_2g_^4^e_g_^2^) one with S = 2 and ^5^T_2g_ spectroscopic term. It needs to be mentioned here that one major difference between the two spin states is the characterization of the HS one by a larger coordination sphere volume due to the occupation of antibonding e_g_* manifold orbitals. Lastly, the rare existence of an intermediate spin state (IS) with S = 1 which is only observed in other coordination polyhedra as prismatic and square planar should be noted [1].

**Figure 1:**
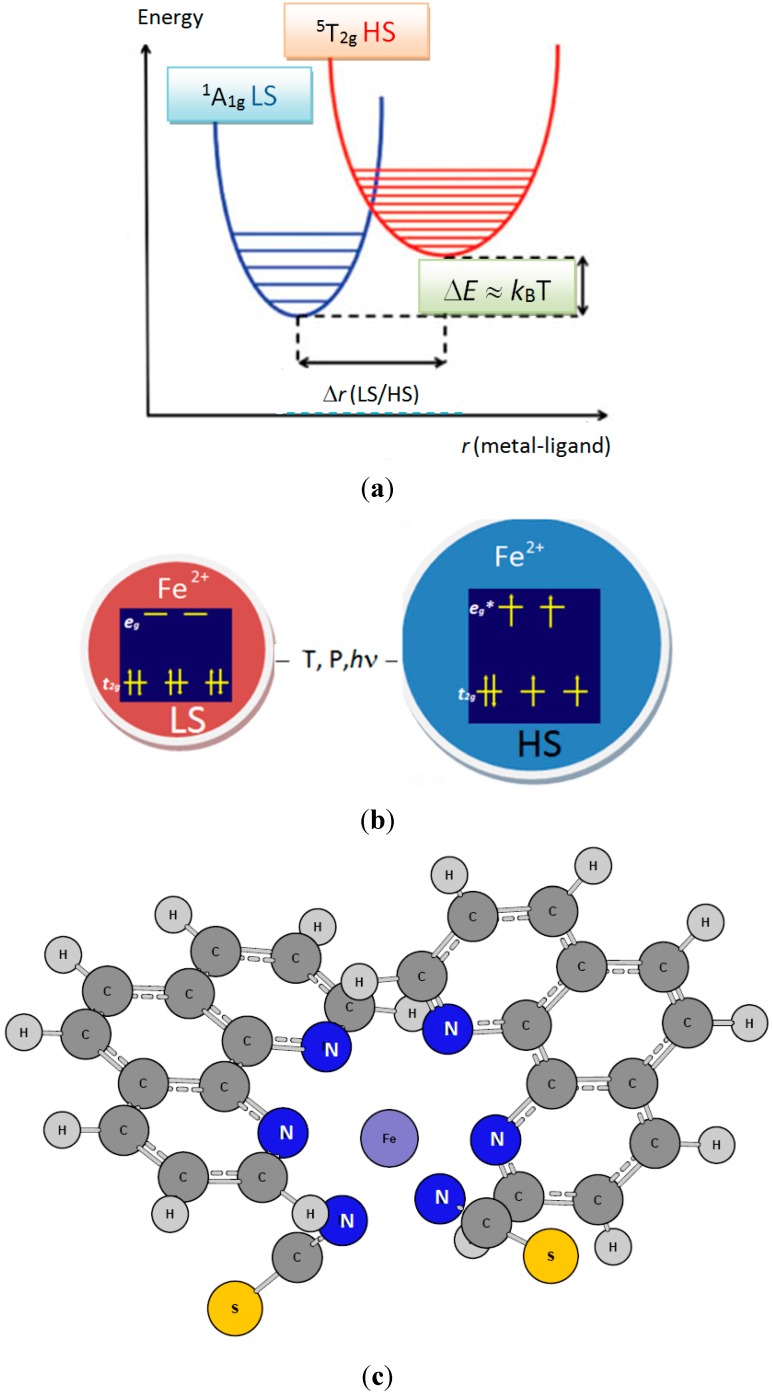
(**a**) Schematic representation of trends of energy *versus* metal-ligand separation in a spin crossover system HS-LS states; (**b**) Relative volumes of HS and LS states with the 3*d*^6^ configuration of Fe^II^ in an octahedral field. The larger HS volume is due to the occupation of antibonding e_g_* orbitals; and (**c**) The molecular structure of [Fe^II^(phen)_2_(NCS)_2_] characterized by two neutral bidentate phen (1,10-phenanthroline) ligands and two charged monodentate NCS^−^ ligands.

The LS/HS bi-stability (Figure 1a,b) is connected with small energy (Δ*E*_HS–LS_ *≈ k*_B_*T* ) magnitudes involved in the switching between these two electronic states so that the transition can be achieved with small perturbations due to external constraints such as temperature, pressure as well as light and electromagnetic fields (T, P, hν, H). A key chemical property is the medium ligand field strength provided by nitrogen-based ligands. In the spectrochemical series comprising unidentate as well as bidentate ligands, listed here below from weak to strong fields, such ligands are found in the middle: I^−^ < Br^−^ < S^2−^ < SCN^−^ < Cl^−^ < NO3^−^ < N^3−^ < F− < OH^−^ < C_2_O_4_^2−^ ≈ H_2_O < NCS^−^ < CH_3_CN < py (pyridine) < NH_3_ < en (ethylenediamine) < bipy (2,2'-bipyridine) < phen (1,10-phenanthroline) < NO^2−^ < PPh_3_ (Triphénylphosphine) < CN^−^ ≈ CO.

In the spectrochemical series of transition ions of the first series divalent iron is also in intermediate position: Mn^2+^ < Ni^2+^ < Co^2+^ < Fe^2**+**^ < V^2+^ < Fe^3+^ < Cr^3+^ < V^3+^ < Co^3+^. Although most works focus on Fe compounds, SCO is also observed with other transition metal ions as discussed by Gütlich and Garcia [2]. Among the numerous spin crossover complexes some are cationic using only neutral ligands, others are neutral while using both neutral and anionic ligands. A typical example is presented in Figure 1c with the [Fe(phen)_2_(NCS)_2_] (phen: 1,10-phenanthroline) complex built from two neutral bidentate phen ligands and two monoanionic monodentate ligands NCS^−^. This is the archetype of Fe(II) SCO compounds synthesized and characterized using X-rays in 1993 by Granier *et al.* [3]. As temperature and light are key parameters to induce the spin-crossover, room temperature bistable compounds are actively sought experimentally.

The SCO phenomenon has been widely investigated by research groups worldwide as well as in our Institute with several synthesized new materials. After more than three decades of investigations, the SCO phenomenon is well understood. From many perspectives, SCO materials development may appear close to applications; a significant number of patents have been deposited, and some research topics concern the ultimate steps before applications [4,5]. In this context, however, one of the main requirements of the scientific community working on SCO is now to get reliable models for this phenomenon [6]. In particular, the absolute necessity to establish a procedure to treat the solid state effects at a quantum mechanical level is noted, while for the isolated molecule, such a description is quite good. For example, besides experimental investigations a current challenge is to enable predicting the properties of the candidate materials exhibiting SCO in a manner accompanying and helping the synthesis procedure, usually heavy and costly. Modeling and theoretical studies are then needed. They are carried out mainly within the well-established quantum theoretical density functional DFT framework [7,8]. However for the treatment of dynamic properties of large systems DFT results can be fed in semi-empirical methods as shown in this paper. Most of the investigations are done at the level of the isolated molecule but, in so far that the physical measurements are done for the solid, further support form computations for the solid-state electronic structure are needed for an account of the whole crystal system. The purpose of this article is to provide those results accounting for the complementary isolated molecule—crystal solid double approach that we label as multiscale study of two selected Fe(II) SCO complexes. It is relevant to mention here that this multiscale aspect also applies to the theoretical levels in use, as developed hereafter.

## 2. Theoretical Framework

In quantum molecular chemistry, the Hartree-Fock (HF) approach [9] presents a large improvement with respect to the Hartree electrostatic model thanks to the introduction of exchange. Exchange (X) is the necessary condition to account for the Pauli Exclusion Principle which rules out the presence of two electrons with the same spin (↑↑ or ↓↓) in the same region of space. As a consequence, in the first half of the last century HF-type calculations have been shown to provide a good description of the molecular orbital and chemical bonding properties in organic chemistry. However correlation (C) is not accounted for in HF; this is the condition which accounts for the behavior of electrons of opposite spins in the same location (↑↓ or ↓↑) and for small molecular systems such a property is accounted for through Configuration Interaction (CI) and highly demanding computational efforts. Nowadays it is becoming well established that calling for DFT framework brings far more accurate results regarding the energetics and related properties. This is because the exchange and correlation (XC) effects are equally treated, albeit at a “local level”, *i.e.*, at the location of the electron which becomes then a “quasi-particle”. In this context far from nuclear physics, the quasi-particle is defined as the electron surrounded by an impenetrable space called “the exchange correlation hole” which consists of merging the Fermi hole (exchange) and the Coulomb hole (correlation). Note that exchange is accounted for in HF in a better way than in DFT because it is done non-locally.

In DFT, XC effects are approximated through different “functionals” such as the local density approximation (LDA) [10] and the generalized gradient approximation (GGA) [11]. Note: In this context the energy *E* is a functional of the electronic density ρ whereas ρ is a function of the position parameter r. Then a functional, in mathematical terms, is “the function (*E*[ρ]) of a function [ρ(r)]”. In DFT one assumes a system with a changing electron density [ρ(r)], and E is a function of that total distribution.

Nevertheless both LDA and GGA are based on local approximations of the electron density. Taking the best out of each one of the HF and DFT solutions led to improvements in *ab initio* molecular calculations with the so-called “hybrid functionals”. They consist of mixing exact exchange following HF (e.g., according to Becke) and DFT-based correlation, as for instance according to Lee, Yang and Parr [12], *i.e.*, the so-called LYP correlation, with proportions that help reproduce physical properties such as the spin state transition. As a consequence most accurate results are found with the so-called hybrid functional “B3LYP” as well as a modified ‘tunable’ one, B3LYP* provided a large—and complete enough—basis set such as all electrons “6-311g(*d*,*p*)” and/or effective core LANL2DZ (*Los Alamos National Laboratory with Double Zeta* polarization) is used. Although several computational methods are known for the molecular chemical compounds, we used in our studies the *Gaussian*09 commercial code [13]. Results of such molecular calculations are needed as a first step for extended solid calculation, especially those of molecular dynamics (MD) as shown in following sections.

At the solid state level, the calculations of the electronic structures were carried out *inter alia* using the VASP package [14]. The interactions between the ions and the electrons are described either with the projector augmented wave (PAW) method [15] or with ultra-soft Vanderbilt pseudo-potentials (US-PP). In the latter approach, the rapid variation of the potential near the nuclei is replaced by substituting the all-electron Hamiltonian with a smoother pseudo-Hamiltonian, which reproduces the valence energy spectrum. The PPs allow a considerable reduction of the necessary number of plane waves per atom for transition metal and first row elements. Thus force and full stress tensor can be easily calculated and used to relax atoms into their ground state. The parallelized calculations are made to run on 4, 8 or 12 processors, according to the size of the structure and the required precision of the calculations through the message passing interface (MPI) procedure. The k-point integration in the Brillouin zone of the reciprocal lattice is carried out with increasing mesh in the successive runs for convergence and relaxation to zero strains. Further details on the different methods and their implications can be found in a recent review paper [16]. Besides the electronic density of states (DOS) we have obtained from the calculations a qualitative picture of the major question raised by chemists: where are the electrons? One answer can be provided through the mapping of the electron localization around the atomic constituents. This can be achieved based on the scheme of the electron localization function (ELF) plots introduced by Becke and Edgecomb [17]. They result from a real space analysis of the electron distribution within the chemical system by comparing it with the free electron gas. ELF is a normalized function which is close to 1 for strongly localized electrons (red spots); 1/2 for the free electron-like behavior (green spots), and close to 0 for no localization (blue zones). Other schemes provide similar results such as the Markovian analysis of electron localization introduced by M. V. Putz in 2008 [18].

## 3. Results and Discussions

### 3.1. The SCO Archetype Compound [Fe(phen)_2_(NCS)_2_]

**Figure 2:**
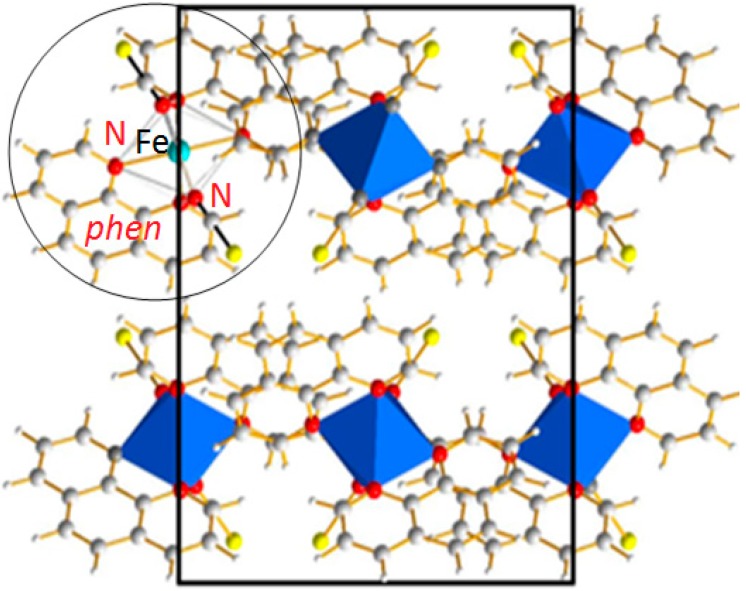
[Fe(phen)_2_(NCS)_2_] SCO complex: Projection of the orthorhombic structure containing 4 formula units. The molecular entity as sketched in Figure 1c is highlighted in the circle.

Most SCO compounds form extended organized solids, meaning that they crystallize in different space groups with long range atomic order. For instance the SCO compound [Fe(phen)_2_(NCS)_2_] adopts an orthorhombic crystal system with four molecular complexes per unit cell. A projection of the crystal packing is given in Figure 2. This compound is one of the most studied among the Fe(II) SCO complexes and is very often cited as the archetype for SCO materials. [Fe(phen)_2_(NCS)_2_] exhibits a first-order phase transition LS → HS reversible in the solid state. The magnetic and photo-magnetic properties reflect an abrupt thermal SCO with *T*_1/2_ = 176 K at which equal HS and LS fractions are found and a narrow thermal hysteresis of Δ*T* ~ 1 K [19] and a complete light-induced SCO characterized by T (LIESST) = 62 K [20]. The abrupt first-order phase transition associated to SCO phenomenon does not affect the symmetry of the crystal packing, being orthorhombic *Pbcn* in both HS and LS, whatever the stimulus used to induce the SCO. At room temperature, the pressure-induced SCO occurs at about 0.6 GPa. The (P, T, light) phase diagram has been experimentally determined by means of variable temperature, pressure X-ray diffraction [21] and photo crystallography [22,23]. Elsewhere this compound has even been used as the model compound to develop photo-crystallographic high-accuracy experimental protocols and, at this occasion, the need for DFT investigation was underlined [24]. [Fe(phen)_2_(NCS)_2_] was also used to investigate the multiscale sequence of the structural modifications associated to the SCO as described above in Section 3.1.

**Figure 3:**
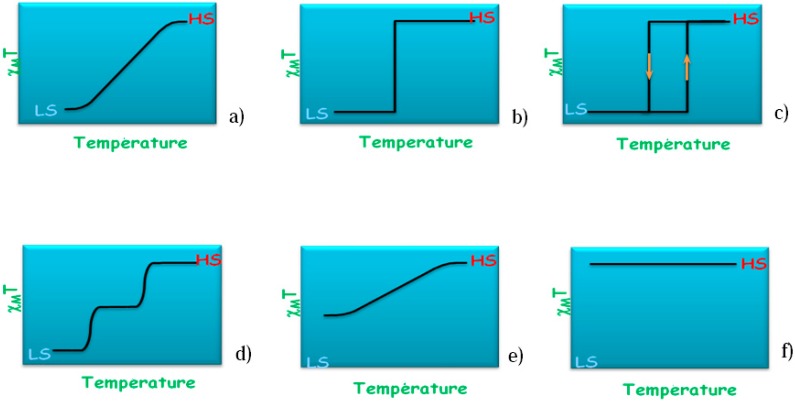
Schematic temperature variation of molar magnetic susceptibility χ_M_ of a SCO compound showing the different types of thermal spin transitions encountered: (**a**) Gradual; (**b**) Abrupt; (**c**) Hysteretic; (**d**) Multistep; (**e**) Incomplete; and (**f**) No transition.

As stated above the thermal transition in SCO compounds can exhibit different behaviors. These scenarios are sketched in Figure 3 where the magnetic susceptibility is plotted against temperature schematically. In the case of the [Fe(phen)_2_(NCS)_2_] SCO complex the behavior is abrupt with a small T hysteresis width as depicted in Figure 3c. During the transition the average Fe–N distance changes from ~1.98 Å (LS) to ~2.20 Å (HS) and the jump between the two spin states occurs at the temperature of *T*_1/2_ = 176 K.

**Figure 4:**
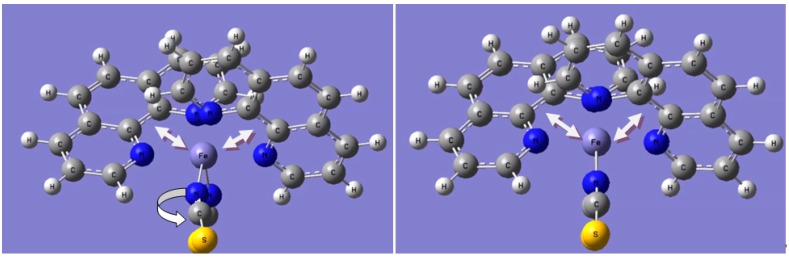
HS [Fe(phen)_2_(NCS)_2_]: Snapshots of the breathing mode of the molecule at 128 cm^−1^ reproducing the experimentally observed mode.

The photo-induced state is characterized by a short lifetime and is subjected to ultrafast photo-switching dynamics. In this context, it is necessary to address the basic mechanisms allowing light to photoswitch a spin-crossover molecular crystal from LS to HS. For this purpose a recent study of [Fe(phen)_2_(NCS)_2_] at T = 140 K (*i.e.*, in the LS state) was done with a femto-second (10^−15^ s) laser pulse at λ = 650 nm leading to identify a LS → HS photo-switching through a metal-to-ligand charge transfer process (MLCT) [25]. A two-step structural trapping occurs: Firstly molecular breathing vibrations are activated but rapidly damped when energy is partly and sequentially transferred to the molecular bending vibrations. This was identified based on time-dependent fast Fourier transform (FFT) of optical transmission showing the activation of a breathing mode at 113 cm^−1^, calculated at 128 cm^−1^ and a delayed activation of a torsion mode at 85 cm^−1^, calculated at 91 cm^−1^. For an illustration, Figure 4 shows two snapshots of the breathing mode with the arrows indicating the remarkable motions of the phen ligands around central Fe. This is also accompanied by a twisting of the two NCS^−^ ligands.

**Figure 5:**
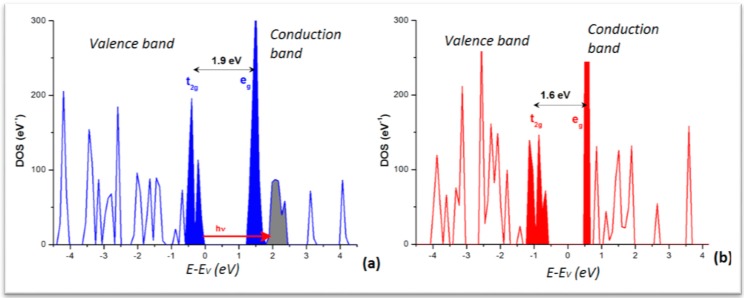
Electronic Density of states (DOS) of [Fe(phen)_2_(NCS)_2_]: (**a**) LS; and (**b**) HS. Energy reference at the top of the valence band (*E*_V_), both varieties being insulators.

From the calculations in the extended crystal, the density of states (DOS) was obtained for the LS and HS phases. The plots are shown in Figure 5 with the major feature of low dispersive and separated DOS peaks in both panels. This is significant of a molecular crystal, as opposed to metals or intermetallics characterized by broader and overlapping DOS. The lower part of the valence band (VB) is dominated by the ligand DOS whereas the VB top and conduction band (CB) bottom are dominated by the transition element Fe *d* states split in the FeN_6_ “nitrogenated” O_h_ field into t_2g_- and e_g_-like manifolds. For the sake of clarity they are shown in solid filled color peaks and separated by the band gap, larger for the LS (~1.9 eV) than for HS (~1.6 eV) as it can be inferred from the crystal field splitting, stronger for LS with empty e_g_ (t_2g_^6^e_g_^0^) *versus* HS (t_2g_^4^e_g_^2^) with occupied e_g_. Note that such gap magnitudes are not to be taken quantitatively in so far that the calculations within DFT regular functionals (local LDA and gradient GGA) do not provide accurate band gaps generally. Nevertheless the relevant information regarding the time-resolved optical studies is the correspondence of the DOS with the MLCT process pointed to above and shown here with the red arrow labeled “hν” going from the blue Fe DOS to the grey ligand’s. Also the DOS explain the stronger absorption of the HS state observed around 760 nm (1.6 eV) as being due to a decrease of the energy gap between t_2g_ and e_g_ bands as the molecular ligand field decreases. This narrowing of the gap, resulting from the longer Fe–N separation, occurs within ~140 fs and correlates well with the 170 fs elongation time obtained by X-ray absorption spectroscopy (XAS). Also in the course of the LS–HS transition, photo-excited singlet ^1^MLCT state (t_2g_^5^e_g_^0^L_1_) and other potential intermediate states such as triplet ones as ^3^T_1_, difficult to identify because of their short-lived lifetimes could not be excluded.

Lastly we point out another feature arising from the solid state calculations regarding total energies. From electronic structure calculations carried out for full geometry optimization of both LS and HS crystal varieties, we find the following total energies: *E*_LS_ = −1081.06 eV; *E*_HS_ = −1078.42 eV, *i.e.*, with a −2.637 eV/cell or ~ −0.7 eV/fu (formula unit) stabilization energy favoring the LS configuration system. This is interesting in view of the ∆*E* ~ −0.95 eV/fu found from molecular calculations [26]. The difference could have technical as well as fundamental origins. For the latter, lattice effects not accounted for in molecular calculations can be invoked. In this context it becomes interesting to see how the electrons are localized within the unit cell containing four formula units.

**Figure 6:**
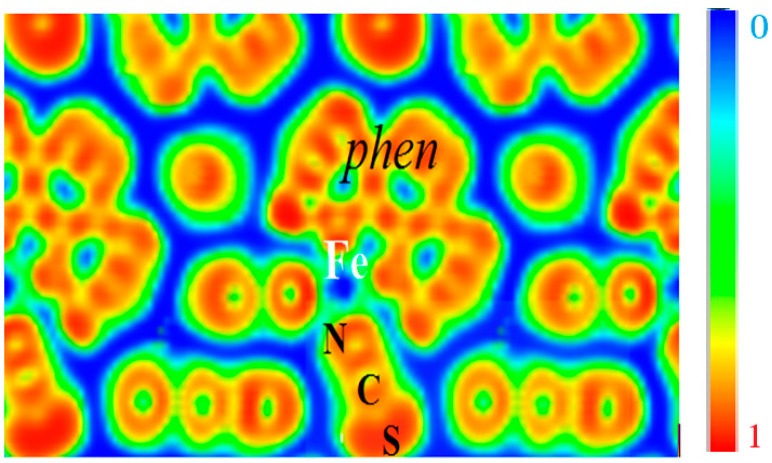
[Fe(phen)_2_(NCS)_2_] SCO complex: The molecular character in the solid state is illustrated by the electron localization function ELF slice dominated by intermolecular blue (zero localization) zones opposite to red intra-molecular zones with ELF = 1. Green areas correspond to free electron gas like medium localization (see text).

This can be visualized from the ELF contours given in Figure 6 which can be seen to closely retrace the molecular entity of the complex with strong localization within the phen and NCS ligands, signaling the bonding within them. A marked feature is the presence of zero localization blue zones around the molecular entities, which allows the supposition that they are isolated from each other. Fe is centered on blue zero localization areas and surrounded by green free electron-like zones connecting with the *N*-terminated ligands. These characteristics are indicative of its positive charge in agreement with expectations of Fe^2+^ ion and of the different Fe–N bonding interactions.

### 3.2. From the Molecule to the Solid: Study of SCO [Fe(PM-BiA)_2_(NCS)_2_]

**Figure 7:**
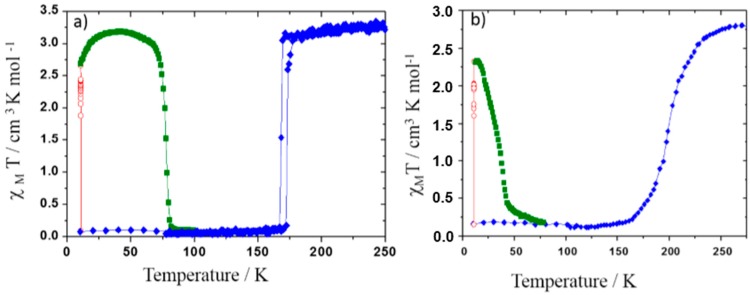
Magnetic properties (molar susceptibility χ_M_) function of temperature in the dark (blue diamond), under 830 nm irradiation (red open diamond) and in temperature in the dark after irradiation (dark green square) of phase I (**a**) and phase II (**b**) of [Fe(PM-BiA)_2_(NCS)_2_].

The [Fe(PM-BiA)_2_(NCS)_2_] compound (PM-BiA stands for *N*-(2-pyridylmethylene)aminobiphenyl) was synthesized in 1997 by Létard *et al.* [27]. It is peculiar among the Fe(II) SCO complexes since it shows polymorphism. Initially, this compound was thought to replace [Fe(phen)_2_(NCS)_2_] as the SCO archetype material but the interplay it subsequently revealed between SCO and polymorphism prevented it to play this role [28]. At room temperature, depending on the synthesis conditions, [Fe(PM-BIA)_2_(NCS)_2_] may crystallize in an orthorhombic *Pccn* or a monoclinic *P*2_1_/*c* space group. Both polymorphs, denoted respectively I and II (Figure 7), exhibit thermal SCO but the former undergoes an abrupt first-order spin transition while the latter undergoes a gradual SCO as shown in Figure 7b. Magnetic and photo-magnetic behaviors as well as the structural studies of these polymorphs have been largely performed and related properties were compared [29] allowing the tracking of the origin of their SCO feature differences. The latter is mainly explained by considerations on the crystal packing. More recently, preliminary experimental investigation of the (P, T) phase diagram by means of neutron diffraction have revealed that applying pressure to the orthorhombic polymorph induces a structural transition to the monoclinic polymorph [30] leading to an intricate spin and structural phases diagram. As it is further shown below, molecular simulation has been used to determine the full (P, T) phase diagram, overcoming the high experimental difficulty to obtain reliable high pressure crystal structure at variable (low) temperature as well as its high cost [31]. In addition, the theoretical investigation allows explaining the very unusual synergy between SCO and polymorphism.

### 3.3. Vibrational Spectroscopy

**Figure 8:**
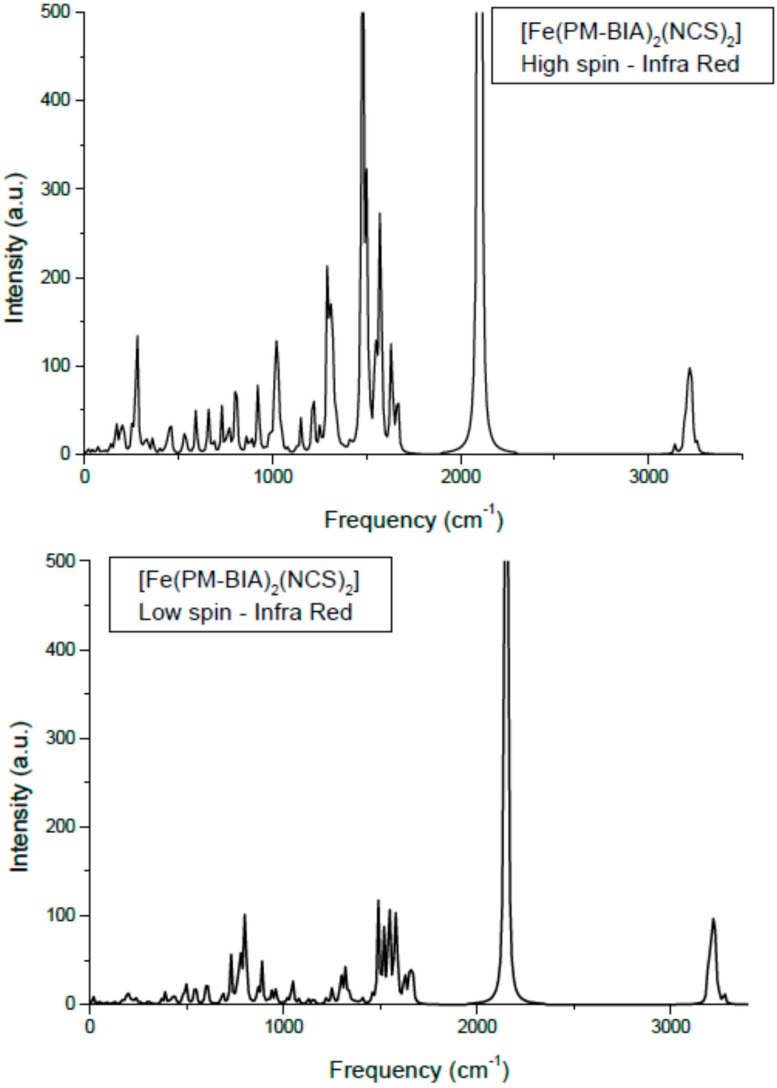
Calculated Infra Red spectra of [Fe(PM-BiA)_2_(NCS)_2_] in the two spin states.

One major signature of SCO complexes is their vibrational spectroscopy mainly through the infra red (IR) and Raman spectra [32,33,34]. Using the hybrid functional B3LYP and an ECP (effective core potential) basis set, LanL2DZ, the molecular structures in LS and HS states are firstly geometrically optimized then the vibration modes are calculated based on the obtained minimum structure. Limiting our results to IR, we show in Figure 8 the spectra for the low spin and the high spin states of phases. Also Table 1 presents the main assignments together with a comparison between the calculated and the experimental frequencies [35]. Although the two spectra show similar shapes and peak positions, the HS IR intensities are higher than LS ones, signaling higher activity in the former state. Also the absence of negative (imaginary) frequencies is a token of reliability for the calculations. The intensity values are relative to the highest value in the set (*i.e.*, follow the intensity of the NC in the NCS band at ~2100 cm^−1^); therefore no reliable relationship to experimental intensities is allowed. Also no scaling was done since the main purpose was to assess the relative energy shifts of the band between HS and LS spin states.

**Table 1:** [Fe(PM-BiA)_2_(NCS)_2_] experimental and calculated IR (infra red) frequencies.

Infra Red ν (cm^−1^)	HS (High Spin)		LS (Low Spin)	
	Experiment	DFT	Experiment	DFT
Fe–N	246	241	342	340
	259	255	364	365
	271	287	368	369
δ(NCS)	438	450	447	426
	469	458	453	442
	478	461	458	446
C–S	Not determined	775	809	768
		779		771
C–N	2074	2030	2124	2123
		2072		2133

The assignments of the different modes: the lower frequency lying modes are the Fe–N stretching appearing at 220–270 cm^−1^ (HS) and 340–400 cm^−1^ (LS). This range corresponds to far-IR. The higher frequency (energy) arises from the average shorter Fe–N in LS (~1.95 Å) *versus* HS (~2.20 Å) states. Bending modes of NCS, δ(NCS) are found centered around 470 cm^−1^ and they show a small dependence with spin state. This is also observed for the C–S elongation around 800 cm^−1^. The intense peaks at ~2000 cm^−1^ are the usual signature of this class of SCO complexes as they are assigned to C–N symmetric and anti-symmetric stretching in the NCS ligand. They are found here at ~2070 cm^−1^ for HS and at higher frequency, ~2120 cm^−1^ for LS.

The calculated frequencies are in good agreement with experiments [35,36]. Some differences may appear due to the fact that the calculations were done on a single molecule; subsequently some missing lattice effects might lead to differences. However quantum mechanical calculations were difficult to achieve due to the huge number of atoms for a four formula unit cell, knowing the already large number of atoms in the molecule ~80. Lastly, we mention that Raman-calculated and experimental results showed similarly relative good agreement. Therefore the DFT-based molecular calculations provide a reliable enough description of the two spin states of a SCO complex, specifically the vibration spectroscopic ones and permit-extracting parameters for use in the following steps pertaining to the solid state investigations.

### 3.4. Semi-Classical Molecular Dynamics

Accounting for intermolecular interactions is necessary for an accurate description of the physcial behavior of an organometallic compound, although such interactions represent a small contribution to the total energy of the chemical system, *i.e.*, with respect to the contributions arising from the intramolecular forces of the isolated molecules. The intermolecular forces act at the macroscopic level; they mainly result from electrostatic and weak van der Waals interactions. Keeping in mind the difficulties of the purely quantum mechanical DFT approach in evaluating these forces [31,35], classical methods like molecular dynamics (MD) ones can be used. In MD methodology the weak van der Waals forces or hydrogen bonding may be modeled within a generalized atom-atom force-field. However this requires the preliminary knowledge of the atomic charges (*q*_i_) and of the potential energy surfaces (PES) deduced from molecular quantum calculations within DFT with its hybrid functionals such as B3LYP [12,13]. The results displayed in Table 2 allow evaluating the force-field parameters. The concomitant combination of DFT-based calculations with molecular dynamics complementarily allows defining an original methodology we call “semi-classical molecular dynamics” (SC-MD).

**Table 2:** [Fe(PM-BiA)_2_(NCS)_2_]: Calculated Mulliken charges *q* of the atoms forming the *Fe–N_6_* octahedron (in *e* units). Atom labels as in Figure 9.

Atomic Species	*q* (LS)	*q* (HS)
Fe	1.5099	1.0442
N_1_	−0.7245	−0.7662
N_2_	−0.7678	−0.6936
N_3_	−0.7567	−0.7326

**Figure 9:**
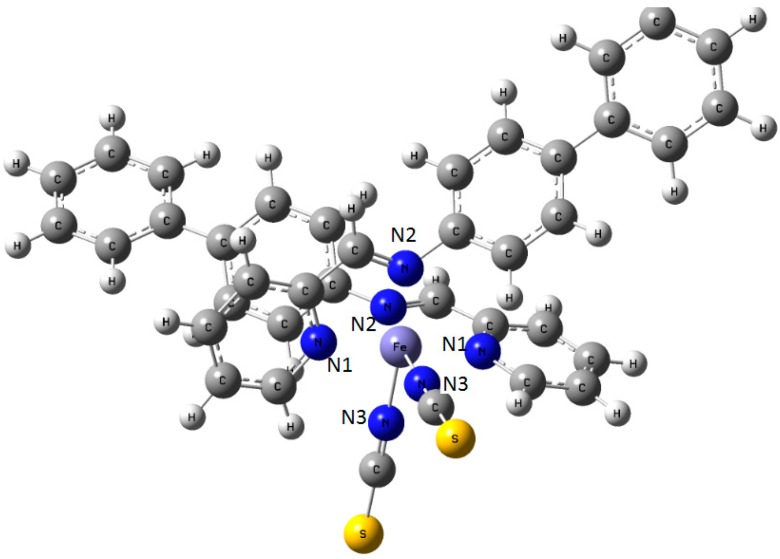
Sketch of the molecule of [Fe(PM-BiA)_2_(NCS)_2_] SCO compound with the labeling of the three different nitrogen atoms in the coordination sphere of central Fe.

Practically, in the classical approach the intramolecular force field includes electrostatic forces between atomic charges, two-body stretching elongations, three-body (3 atoms) bending forces, as well as dihedral interactions involving four centers. Using the hybrid B3LYP functional, with effective core potential basis set ECP-LANL2DZ, the resulting optimized molecular structure and point charges allow generating the PES. The curve is then fitted with Morse potential *V*_Fe–N_ and harmonic *V*_Fe–C–N_ potentials, by varying alternatively one of the three Fe–N_i_ distances of the molecule. The Morse expression of short range potentials is written as:

(1) $$V_{Morse}(r_{ij})=E_{ij}^{0}[\{1-\text{exp}(-\rho_{ij}(r_{ij}-r_{ij}^{0}){)\}}^{2}-1]$$

where *E_ij_* is the potential depth, ρ*_ij_* the electronic hardness and *r_ij_* the equilibrium distance between two *i* and *j* species. Following other studies a constant values of ρ*_ij_* = 1.33 Å^−1^ was used independently from the nature of *N_i_* and of the spin state. The three types of nitrogen (*i* = 3) are depicted for the molecular entity in Figure 9. In fact, using a constant value of ρ*_ij_* is generally adopted in classical force field studies even if the coordination polyhedra around a specific atom *i* are of different types and strongly distorted [31].

In a second step, MD simulations are performed including the intramolecular interactions at the level of the molecule. The parameters are adjusted with respect to the molecular geometry and the spectroscopic properties by using the DL_POLY MD code [37] which yields a molecular field for the HS and LS spin states.

To finalize, the generalized force fields are applied to the complex in its extended crystal lattice: After a minimization step at ΔT = 1 K for both spin states, MD simulations are performed stepwise up to 300 K; the same protocol is followed for the cooling procedure. Best van der Waals parameters are searched for, in order to reproduce structures both in the LS state (25 and 140 K) and HS state (298 K) [38]. With these final intermolecular force fields, the complete set of runs allow evaluating respectively *T*_1/2_ ↑ and *T*_1/2_ ↓ and transition enthalpy Δ*H* (LS → HS) at *T*_1/2_, the so-called Δ*H*_1/2_. *T*_1/2_ and Δ*H*_1/2_ are related, as shown in a phenomenological thermodynamics view, by modeling the LS-HS domain mixture as a regular solid-solution. This domain mixture arising along the SCO, typical from a first-order phase transition in a crystal, has been observed by means of variable temperature X-ray Diffraction (XRD) [29].

As a result of the above protocols applied to the [Fe(PM-BiA)_2_(NCS)_2_] complex, the fitted potentials from quantum *Gaussian*09 calculations combined with van der Waals interactions, allow the acquisition of the vibration modes (I.R. and Raman) of the molecule in its two spin states by molecular simulation (Figure 8). Particularly we found, in agreement with the experiment [30], that the *Fe–N_6_* octahedron is more distorted in the high-spin state than in the low-spin one, as illustrated by the distance changes: Δ*d*_HS_ = 0.21 Å much larger than Δ*d*_LS_ = 0.03 Å as well as for the angles extremes: Δθ_HS_ = 26.4° > Δθ_LS_ = 13.7°.

Transferring the obtained fields to the solid structure, the simulations provide the inputs applied to the orthorhombic crystal lattice. Intermolecular interactions show evidence of hydrogen bonding between the NCS ligand at the sulfur end atom with nearest neighbors hydrogen belonging to the aromatic cycles. This feature has been noted as one of the most specific ones of the derived complexes family of general formula [Fe(PM-L)_2_(NCS)_2_] where L is an aromatic ligand. The evolution of the structure with temperature shows that the LS → HS transition occurs at *T*_1/2_ ↑ = 120 K which is 50 K lower than the experimental value. The change of the transition enthalpy, Δ^MD^*H*_1/2_ = 14 ± 2 kJ/mol corresponds to the transition temperature *T*_1/2_ = 150 K. Experimental measurements give Δ*H*_LS → HS_ ~11 kJ/mol. This allows casting confidence on the calculation procedure. We note however that the departure from the experimental value is likely due to the drawbacks of using a semi-classical model in view of the probable prevalence of quantum effects below 100 K.

**Figure 10:**
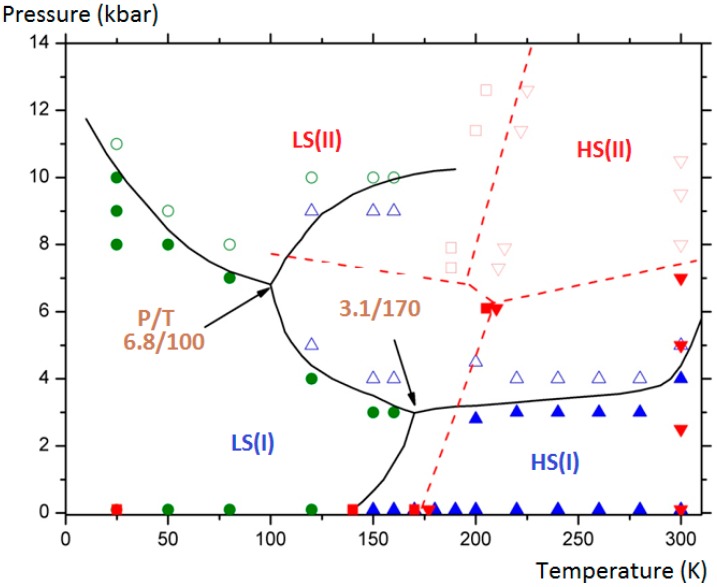
[Fe(PM-BiA)_2_(NCS)_2_]: (P, T) phase diagram showing four solid phases: LS (I): filled green circles; HS (I): filled blue triangles; LS (II): empty green circles; HS (II): empty blue triangles). The domains delimitated by straight curves generate two triple points. Experimental points from [30] are presented for comparison (LS (I): filled red squares; HS (I): filled red down triangles; LS (II): empty red square; HS (II): empty red up triangles).

The final aim of the calculations was to assess and complete the experimental (P, T) phase diagram of the compound by showing the domains of existence of the monoclinic polymorph (II) in its two spin states (LS/HS). Furthermore the delimitation of the different domains allowed generating two triple points. The results are depicted in Figure 10 where the connected lines are drawn as a guide for the eye: for LS (I), HS (I) and HS (II) equilibrium: at T = 170 K and P = 3.1 kbar; for LS (I), HS (I) and HS (II) equilibrium, at T = 100 K and P = 6.8 kbar.

It needs to be stressed that subsequent experimental investigations at low temperature under high pressure by means of neutron diffraction have confirmed the predicted phase diagram (Figure 10). Indeed the possible HS (I) to LS (II) and HS (II) to LS (I) switching by applying high pressure at variable temperature was demonstrated. Experimental difficulties to extract accurate structural data as well as an accurate determination of the effective sample temperature have prevented covering the whole (P, T) phase diagram. The molecular simulations, once validated by some experimental points as performed here, appear to be a predominant tool to investigate the full phase diagrams. We also note that the behavior of this sample is probably even more complicated than described here since diffuse reflectance investigations under high pressure have revealed that a third phase could be obtained at moderated pressure. The localization of this new phase is not clearly assigned either to the surface solely or to the whole bulk material. Comparative investigations of surface and volume behaviors are still a challenge in both the multiscale experimental and theoretical approaches.

## 4. Basic Experimental Tools

Among many other experimental approaches, the determination of the magnetic properties and of the structural characteristics is at the heart of the multi-scale SCO phenomenon understanding. The synergy between the experimental and theoretical approaches is particularly efficient in these cases.

### 4.1. Multi-Scale X-ray Diffraction Approach

**Figure 11:**
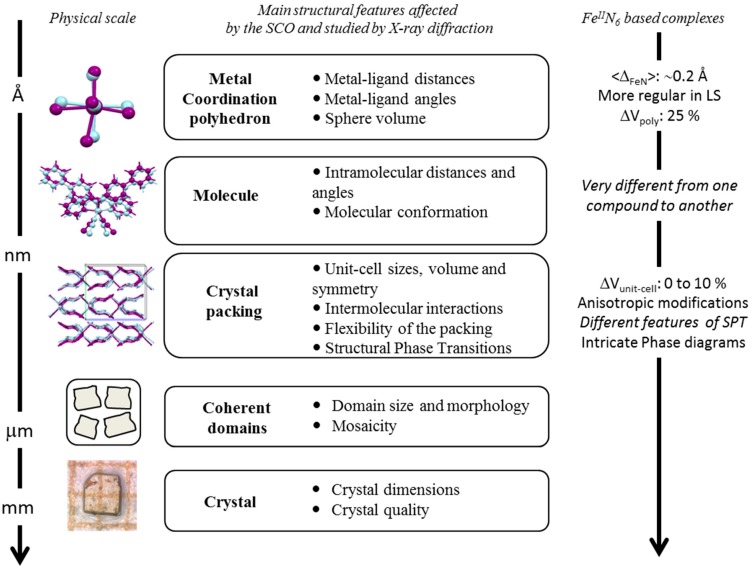
Scheme of the multiscale structural effects of the SCO probed by X-ray diffraction including some general trends for Fe(II)N_6_ based complexes.

The SCO phenomenon is easily observable by X-ray diffraction since the modification of the electronic configuration induces a significant shortening of the metal-ligand distances from HS to LS. The determination of the structural properties is considered as a mandatory step in the investigation of SCO materials since the early stage of this field. Through these decades, the structural approach has allowed a more deep understanding of the SCO phenomenon in the crystalline state but also reveals the richness of the associated SCO mechanisms. Many published reviews are focusing on the latter, including structure-properties relationships [39,40], pressure effects [41,42], structural and phase transitions interplay [43], time-dependence mechanisms [44] or non-periodic situations [45,46] for instance. The important point to highlight here is that this experimental approach gives rise to a multi-scale description of the SCO phenomenon, from the atomic to the macroscopic physical-scales (Figure 11).

At the scale of the metal coordination sphere, the SCO corresponds to a modification of the metal-ligand bond lengths that induces a volume reduction (from HS to LS) of always the same value, whatever the stimulus used to provoke the SCO, nearly 25% in the case of FeN_6_ environments. It is important to note that the SCO is also accompanied, in many cases, by a distortion of the metal-ligand polyhedron. The lengths and distortions modifications inside the coordination sphere can be related to the molecular breathing and bending vibrations modes [25] (Figure 4). The propagation of these modifications to the molecular scale then to the intermolecular scale result in a breathing of the crystal packing showing a large range of amplitude, from about 0% to 10%. At an upper scale, typically at the coherent domain one, experimental investigations are still underway in the community to describe properly the effects of the SCO phenomenon [40]. Among the other present experimental challenges is the access to the full phase diagram, since for example reliable structural data under pressure at variable temperature are yet difficult to obtain on molecular crystals. As shown in this paper, theoretical approaches can efficiently complete the experimental data on this crucial aspect.

### 4.2. SCO Evidenced by Magnetic Measurements

As already specified above, the SCO for Fe(II) ions in an octahedral environment corresponds to a switch from the t_2g_^6^e_g_^0^ (LS) state to the t_2g_^4^e_g_^2^ (HS) leading to a magnetic transition from diamagnetic to paramagnetic behaviors. Due to the strong contrast shown by the latter, the experimental observation of the magnetic properties, generally performed using a Squid magnetometer, is commonly used as the initial and main characterization method performed on a material likely to present a SCO [32]. Nowadays, magnetic measurements allow following the thermo- and light-induced SCO almost routinely, while the pressure-induced SCO still requires more instrumental development in order to be easily accessible. This experimental approach probes the whole volume of the sample and therefore the switch that occurs at the molecular level is seen at the physical scale of the crystals used for the measurement. In other words, the observed magnetic response can be seen as an average of the true local situations. At the molecular level, there are obviously only two possible responses, diamagnetic (HS) or paramagnetic (LS), while at the sample scale it is the ratio between the HS and the LS states that give the magnetic response. An archetype example of magnetic measurements is given in Figure 3. As a consequence, when converting from 100% HS (0% LS) to 0% HS (100% LS), magnetic measurements give access to the spin state ratio modification. This kind of measurement allows determining the characteristic temperature, T_½_, at which equal populations of HS and LS are found for the sample, as well as various useful parameters such as the limit temperature of photo-inscription, namely the T (LIESST) (Light-Induced Excited Spin-State Trapping) obtained after photo-irradiation at low temperature. The richness of the SCO phenomenon leads to a wide diversity of magnetic transition features: abrupt, abrupt with hysteresis, gradual, incomplete, or with multi-steps (Figure 3). One of the main goals of the SCO investigation through a wide panel of techniques, extensively reviewed elsewhere [33,34] is to understand these behaviors in order to subsequently and rationally control the SCO features. As shown below, the multiscale theoretical investigations may contribute to the understanding of these various behaviors, notably the light-induced ones.

## 5. Conclusions

In this review we have shown the relevance of a multiscale approach of a particularly interesting class of transition metal complexes, the spin cross-over SCO compounds, going from the level of the individual chemical molecule to the range of the extended ordered solid. The chemical scale approach also relies on theoretical modeling tools considered at different (multiscale) levels of sophistications. The latter are mainly based on the density functional theory around which different methods are built such as those devoted to the treatment of the isolated molecule *versus* those which treat the whole crystal solid. Also we have shown that the purely quantum level is far too difficult to use when molecular dynamics MD results are sought and semi-classical MD methodology is then called for.

The two SCO examples were chosen so that a broad span of properties is obtained in an illustrative manner for the benefit of the reader. In [Fe(phen)_2_(NCS)_2_] the results obtained from the vibration frequencies of the molecule were used to assess the LIESST photoexcited states pertaining to the internal breathing and torsion modes in the solid. Also, for the crystal compound, the electronic density of states (DOS) and the electron localization function (ELF) suggest a molecular behavior preserved in the solid state. In the [Fe(PM-BiA)_2_(NCS)_2_] molecule, the validation of the calculated IR and Raman frequencies *versus* experiment led to the establishment of potential energy surfaces fitted with semi-empirical potentials in order to facilitate the MD calculations. These have led to complete the experimental (P, T) phase diagram.
